# The Y factor in the cardiac syndrome X

**DOI:** 10.1007/s12471-012-0304-8

**Published:** 2012-07-19

**Authors:** Y. Appelman

**Affiliations:** Department of Interventional Cardiology, VU Universitiy Medical Center, De Boelelaan 1117, 1081 HV Amsterdam, the Netherlands

What is interesting in a patient with chest pain and normal coronary arteries on the angiogram? Not much for the Y factor, especially as there is not much we can see, investigate, measure or treat. The publication by Vermeltfoort et al. in this issue is very reassuring [1]. The group in Tilburg in collaboration with the VU University Medical Center in Amsterdam did an excellent job in highlighting the recent data on the prognosis of patients with cardiac syndrome X.

The term cardiac syndrome X was first used in 1973 to describe a condition that to this day still remains a bit of a mystery [2]. Defining cardiac syndrome X is difficult as there is still no real agreement on a definition. The diagnosis is generally made when angina-like chest pain, a positive response to stress testing and angiographically normal coronary arteries are present. Over the years, many theories have been discussed [3]. Syndrome X is currently accepted to be a heterogeneous clinical diagnosis which includes genetic, coronary microvascular, metabolic, hormonal and cardiovascular risk factors.

Vermeltfoort et al. observed and reported in this Journal that there is no markedly increased mortality within 5 years after making the diagnosis, only 1.5 % [1]. Yet the average age of the population is around 55 years and in the largest study included in the analysis (Women’s Ischemia Syndrome Evaluation: WISE study, *n* = 318)) there was a 2.5 fold increase in mortality in cardiac syndrome X patients, compared with a control population [4]. Also the number of cardiovascular events reported is considered to be small in the meta analysis, but that is partly related to disregarding left ventricular dysfunction as a relevant endpoint. In the WISE cohort there was a tenfold increase in women admitted for cardiac failure compared with controls in 5 years. A significant increase in events has also been found in other studies, as reflected by the 5 % cardiovascular event rate reported in the meta analysis.

What is very striking is the high rate of ongoing and recurrent symptoms in syndrome X patients. Apparently they are hard or impossible to treat and it seems difficult to make them symptom free.

There is always room for improvement. It is crucial to know in how many patients ischaemia has been really proven prior to the coronary angiogram procedure. Was ischaemia detection only based on ECG changes, complaints or METS score during the stress test? This is even more relevant if the data from the WISE study are again used. Patients with cardiac syndrome X and exercise-induced, MRI-detected ischaemia had an event rate of 43 % within 3 years. Such a high event rate in a subgroup of patients would suggest that it should be feasible to identify a specific high-risk group. In this high-risk group, short-term pharmacological studies would be feasible to demonstrate efficacy.

Another piece of information the authors could look into is the X-Y story. The variation in male and female patients goes from 0 % in some studies to 100 % in others. It is essential to also perform the analysis based on gender, as conflicting data exist with respect to cardiovascular outcome. There could be fundamental differences in diagnosis, treatment, prognosis and underlying mechanisms, especially in the light of the interesting paradox in female patients who have a higher frequency of normal angiogram, less extensive coronary artery disease, higher prevalence of angina and worse clinical outcome [5].

Should we redefine the cardiac syndrome X?

There are two options on how to proceed in this patient group. If the coronary angiogram is normal, without any sign of coronary disease and in absence of calcifications, it would be of interest to perform an invasive coronary flow reserve (CFR) measurement to rule out microvascular disease. An alternative strategy could be to perform more profound ischaemia detection through magnetic resonance imaging perfusion or positron emission tomography perfusion with CFR added following the coronary angiogram to show subendocardial hypoperfusion. The distinction between microvascular disease and lack of disease can be assessed with these diagnostic modalities. Subsequently, the concrete diagnosis should be translated into adequate treatment where several drugs can be considered that improve endothelial function [6].

The extra step in the diagnostic approach is mandatory to reduce symptoms and improve prognosis to match the prognosis of the ‘healthy’ population, as was measured in the WISE study where only female patients were included. Therefore, the proposed larger prospective study by Vermeltfoort seems a logical step in unravelling the Y of the syndrome X mystery.
